# Incongruent active head rotations increase visual motion detection thresholds

**DOI:** 10.1093/nc/niae019

**Published:** 2024-05-16

**Authors:** Kate Pickard, Matthew J Davidson, Sujin Kim, David Alais

**Affiliations:** School of Psychology, The University of Sydney, Sydney, NSW 2006, Australia; School of Psychology, The University of Sydney, Sydney, NSW 2006, Australia; School of Psychology, The University of Sydney, Sydney, NSW 2006, Australia; School of Psychology, The University of Sydney, Sydney, NSW 2006, Australia

**Keywords:** active perception, multisensory, visual motion, self-motion, thresholds, bias

## Abstract

Attributing a visual motion signal to its correct source—be that external object motion, self-motion, or some combination of both—seems effortless, and yet often involves disentangling a complex web of motion signals. Existing literature focuses on either translational motion (heading) or eye movements, leaving much to be learnt about the influence of a wider range of self-motions, such as active head rotations, on visual motion perception. This study investigated how active head rotations affect visual motion detection thresholds, comparing conditions where visual motion and head-turn direction were either congruent or incongruent. Participants judged the direction of a visual motion stimulus while rotating their head or remaining stationary, using a fixation-locked Virtual Reality display with integrated head-movement recordings. Thresholds to perceive visual motion were higher in both active-head rotation conditions compared to stationary, though no differences were found between congruent or incongruent conditions. Participants also showed a significant bias to report seeing visual motion travelling in the same direction as the head rotation. Together, these results demonstrate active head rotations increase visual motion perceptual thresholds, particularly in cases of incongruent visual and active vestibular stimulation.

## Introduction

Merging multiple streams of information from across different sensory systems results in a more robust perception of the world around us (Ernst and Banks 2002, Alais and Burr 2004, Ernst and Bülthoff 2004, Alais et al. 2010). A key example is combining vestibular self-motion information with visual motion signals to correctly interpret the source and attributes of both our own self-motion and distal motion in the environment (DeAngelis and Angelaki 2012, Cullen 2012). However, many questions remain regarding how self-motion signals are accounted for such that an accurate and robust perception of object motion can be maintained. One key open question is how natural and realistic vestibular signals may contribute to visual perception of motion on the retina.

A well-studied example of visual and vestibular motion interaction is the study of optic flow and heading perception (e.g. Gu et al. 2006, reviewed in DeAngelis and Angelaki 2012). Heading refers to the perceived direction of one’s translation, with the resulting global visual motion signal arising from relative motion of objects in the scene known as optic flow (Britton and Arshad 2019, Zeng et al. 2023). Subtle changes in either the physical heading direction or cued direction from optic flow can be manipulated to determine overall perception of heading direction (see Britton and Arshad 2019 for a review). Studies of macaque monkeys suggest integration of these signals involves visual-vestibular cells of the dorsal aspect of the medial superior temporal area (MSTd), where cells tuned to congruent translational movement (visual-vestibular direction preferences are aligned), or with opposite tunings occur in roughly equal proportions (Gu et al. 2006). While these cells are mostly reliant on visual cues when visual signals are strong (e.g. when measured in response to tasks with 100% visual motion coherence) (Gu et al. 2006), degrading the visual signal leads to an upweighting of vestibular input to maintain accurate perception (Morgan et al. 2008), with the congruent cells in particular aiding discrimination of small changes in heading direction (Gu et al. 2006, Takahashi et al. 2007, MacNeilage et al. 2012). In contrast to the findings for translation, Takahashi et al. (2007) found only oppositely tuned rotational cells in the MSTd. While congruent optic flow and heading movements occur regularly when moving through the world, and we would therefore benefit from a system designed to enhance congruent visual-vestibular signals in order to aid navigation, rotational movements of the head and body result in visual motion opposite to the direction of rotation, which must be accounted for in order to maintain robust motion perception (Takahashi et al. 2007). It has therefore been hypothesized that the role of the oppositely tuned rotational cells in the MSTd is to disambiguate confounding effects of optic flow arising from self-rotation across a stationary world (Takahashi et al. 2007, Morgan et al. 2008) by providing an exafferent signal to be subtracted from the sensory response.

In these examples, the underlying neurophysiology for both translational and rotational motion systems is serving to maximize veridical motion perception in visual-vestibular conditions. However, just how active rotational head movements might affect thresholds for visual motion perception remains unknown. This is important to understand, as though both translational and rotational movements of the head occur when navigating the world everyday, rotations are far more frequent and common behaviours and fundamental to tracking objects or exploring a visual environment (Spering et al. 2011). Here, we directly contrasted the visual motion thresholds required to maintain ∼75% accuracy on a motion detection task during active head rotation. In two experiments, we contrasted left/right motion detection thresholds with congruent and incongruent head rotations, as well as stationary conditions. We predicted that as both incongruent and congruent conditions may require increases in processing and attention due to the added self-motion signals, these motion thresholds should be higher than stationary thresholds (Kinchla 1992, Lavie et al. 2004, Swallow and Jiang 2013, Heinrich et al. 2020). When comparing congruent and incongruent conditions, we predicted lower visual motion thresholds for congruent than incongruent rotations for two reasons. First, it is proposed that there is a sensory prior that the distal world is stationary (Weiss et al. 2002, Welchman et al. 2008, Freeman et al. 2010), and thus incongruent visual motion signals would be more likely attributed to a stationary object or background. A system designed to disambiguate optic flow arising from rotation, possibly driven by incongruent visual-vestibular cells (Takahashi et al. 2007, Morgan et al. 2008), could suppress these expected incongruent motion signals (thus raising thresholds) in order to highlight any independent motion in a scene. Second, as smooth-pursuit tracking is a common behaviour to gain as much information as possible about a moving object (Spering et al. 2011), we reasoned that congruent head rotations would similarly improve congruent motion-detection thresholds. While most previous research on tracking has focused on smooth-pursuit eye movements, MacNeilage et al. (2012) found object discrimination thresholds were significantly lower when accompanied by congruent vestibular cues, compared to just visual cues from optic flow. Together, this suggests that tracking a moving object via congruent head rotations should aid visual perception.

The current experiments test these predictions using a virtual reality headset to maintain control of the visual environment and to record head speed and rotation with high temporal resolution. To preview the results, we found increased visual motion thresholds for rotation conditions relative to stationary. There was some evidence for increased thresholds for incongruent rotation conditions when compared to both congruent and stationary conditions, though this result could be driven by participants’ bias to respond in line with the direction of head rotation.

## General methods

### Apparatus

A Vive Pro Eye head-mounted display (HMD) was used to present the stimulus. The HMD had a resolution of 2880 × 1600 pixels (1440 × 1600 per eye), a refresh rate of 90 Hz, and a horizontal field of view (FOV) of approximately 110°. The virtual environment and stimuli were rendered with an NVIDIA GeForce RTX 2080 Ti graphic card and programmed in Unity version 2020.2.4f1 using the SteamVR Unity plugin (v1.21.12).

### Data analysis

All data were processed and visualized using Matlab R2022a. Statistical analyses were performed in JASP 0.17.1.0 and Matlab. Cumulative Gaussian psychometric functions were fitted to the data for each condition and participant using the maximum likelihood method (Watson 1979), with a guess rate of 0.5. Individual participant signal-intensities required to achieve 75% accuracy were taken from their psychometric functions, and the mean of participants was used as our group measure of thresholds for visual motion perception in different conditions.

### Linear mixed-effects analysis

We performed linear mixed-effects (LME) analyses to determine whether direction and congruence of visual-active vestibular signals had a significant effect on visual motion detection thresholds. Specifically, we performed linear mixed-effects model (LME) comparison in Matlab (fitlme.m), which fits models using the maximum likelihood method. We compared a full model with fixed-effects for congruence and stimulus-direction, their interaction, and random intercepts per participant [Thresholds – Congruency * Stimulus Direction + (1 | Participant)], with a restricted model without the interaction term [Thresholds – Congruency + Stimulus Direction + (1|Participant)], and a model of just random effects per participant [Thresholds – 1 + (1|Participant)]. The goodness of fit was compared for each model (in increasing complexity) in a stepwise manner using likelihood ratio tests, and significance reported when the goodness of fit significantly improved upon the alternate model. We report the result of the likelihood ratio test and associated *P*-value of model improvement. We note that fixed effects coefficients for the levels of a categorical variable are calculated relative to an arbitrary reference (e.g. Stationary vs Congruent). When reporting on significant model improvement, we report these fixed effects comparisons as coefficients (β), their 95% confidence intervals (CIs), and difference contrasts (*t*-statistics).

### Exclusion criteria

An adaptive staircase procedure (Quest: Watson and Pelli 1983) controlled the strength of the motion stimulus and maintained performance at 75% accuracy. Staircases from two sessions of six motion conditions (left/right motion directions × left/right/stationary rotation conditions) were plotted and inspected and any participant not attaining the required 75% accuracy threshold had their data for that condition removed. Trials across the two sessions were then combined to generate one psychometric function (PMF) for each of the six motion conditions for each participant. The means of these PMFs were the dependent measure of threshold.

## Experiment 1

**Figure 1.**
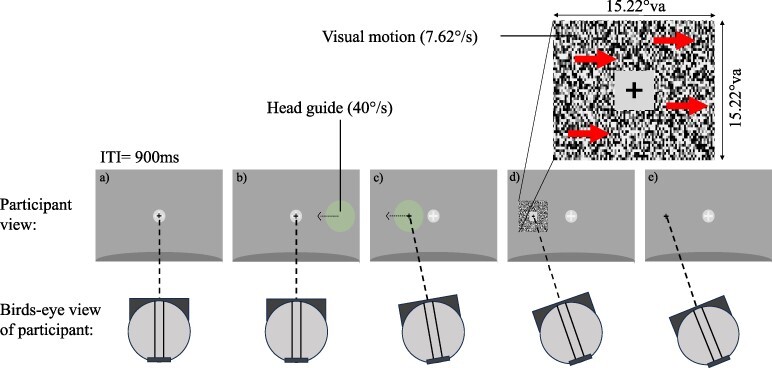
Example trial sequence with incongruent head rotation. (a) In VR, participants aligned their head-centred fixation cross with a world-centred forward marker (grey circular region with cross). (b) Upon fixation, a coloured motion-guide entered their periphery and translated horizontally across the screen with a speed of 40°/s, indicating the direction and speed of the required head rotation. Participants were instructed to monitor the guide as it approached their fixation point and prepare to make a head turn to follow it while keeping their head-centred fixation cross in the centre of the motion guide. (c) The guide reached the world-centred marker after 1 s, which was when participants initiated a head turn to keep their head-centred fixation cross on the motion-guide and their eyes on the head-centred fixation cross. The head-centred cross provided live feedback as to how closely they were following the guide. The motion-stimulus would appear after 1.2 s, so 200 ms after beginning the head rotation. Arrows were not present but represent an example direction of motion. (d) Participants indicated the perceived direction of retinal motion using the left and right arrow keys, then returned to centre for the next trial (not shown), with an inter-trial interval (ITI) of 900 ms. The motion signal consisted of a black and white noise pattern drifting left or right, overlaid by stationary black and white noise, with the contrast ratio controlled by the adaptive staircase to maintain 75% correct performance. The speed of the retinal motion was 7.62°/s. View a short video of the procedure at https://osf.io/nf4uh and https://osf.io/r5t4e

The first experiment measured visual motion detection thresholds by varying signal-to-noise ratio while observers were either stationary or making a head rotation that was congruent or incongruent with the direction of visual retinal motion. Head rotation direction and speed was controlled by having subjects pursue a motion guide (see Fig. 1).

### Methods

#### Participants

Twenty-six individuals were recruited for this experiment. Six were removed from analysis due to technical issues during data collection (*n =* 2), incorrectly understanding task instructions (*n =* 3), or incorrectly performing rotations (*n =* 1), reducing the final number for analysis to 20. Participants had corrected-to-normal vision and no neck or vestibular conditions. They were recruited from the University of Sydney undergraduate Psychology cohort, with one participant a naive member of the authors’ laboratory. All participants were naïve to the purposes of the experiment and gave informed consent prior to participation. The study protocol was approved by the University of Sydney Human Research Ethics Committee (HREC 2021/048).

#### Stimuli

The motion signal consisted of a translating square aperture containing a patch of random luminance pixels whose average contrast (signal intensity) was controlled by an adaptive staircase (Quest: Watson and Pelli 1983) to maintain performance at 75%. This pattern of 120 × 120 pixels would drift left or right at a speed of 7.62°/s within the square aperture. The motion signal was generated using a luminance signal-to-noise ratio (LSNR) where varying the signal-to-noise ratio altered the strength of the motion signal while the mean contrast remained constant (van der Smagt and van de Grind 1999, Lankheet et al. 2002). The contrast value of individual pixels was drawn from a normal-distribution between 0 and 1, and their position was updated at 2 pixels a frame. The noise pattern was also 120 × 120 pixels, with a normal distribution of contrast values per pixel. The average contrast of the noise pixels was adjusted using the formula Contrast_noise_ = sqrt(1 – Contrast_signal_^2^). As the adaptive staircase adjusted the contrast of the motion pattern, the motion and noise pixels were combined on each presentation frame by taking their average value per pixel. The noise was updated in synchrony with the movement of the motion signal, ensuring the noise and moving pattern could not be visually segregated based on temporal differences (Lankheet et al. 2002).

The aperture subtended 15.22° × 15.22° and was locked to participants’ head position such that when a participant turned their head, the aperture containing the stimulus always remained in the centre of participants’ vision and with the same relative orientation. The movement of the aperture directly reflected the movement of a participant’s head and as a result, the retinally defined speed within the aperture was fixed at 7.62°/s. Consequently, there were no eye movements necessary to follow the stimulus and the stimulus aperture was not moving across the retina. A fixation cross of 1.2 °va surrounded by a grey square occupying 7.15 °va was presented in the centre of the stimulus to aid steady fixation and limit retinal motion stemming from confounding eye movements. A video of the stimulus and procedure is available at https://osf.io/nf4uh and https://osf.io/r5t4e.

#### Procedure

Participants always saw a head-centred fixation cross which they could align with a world-centred forward marker so that each trial began from a central position. A circular head-guide was used to indicate required head movement. If the head guide was presented centrally, participants would keep their head stationary. Otherwise, the guide would enter from the edge of the display, travelling in a three-dimensional (3D) arc from one side of the display to the other at 40°/s, serving as a cue for the upcoming direction of rotation. Participants were instructed to start turning their head to follow the guide once it arrived in front of them at the fixation point, which was after 1 s. The guide would be replaced by the stimulus after 1.2 s, so 200 ms after participants began rotation (see Fig. 1). The stimulus was presented for 400 ms. Participants would then press the left or right arrow key to indicate perceived direction of visual motion seen within the aperture, before returning their head to centre for the next trial. Participants completed a practice version of the experiment to coordinate following the head guide and responding to the stimulus. The practice experiment consisted of 30 trials in a fixed order without feedback (10 stationary, 10 with congruent motion, and 10 with incongruent motion, with equal split of motion travelling left or right within each condition). In the subsequent experiment, each participant completed two blocks of 180 trials each (2 stimulus directions × 3 head rotations × 30 trials per condition) giving a total of 360 trials per participant and all trial types were randomly interleaved within a block.

There were six visual-active vestibular motion signal combinations of interest in the current experiment, those being left–left, right–right, left–right, right–left, left–stationary, and right–stationary (with the first in the pair referring to visual stimulus direction and the second head rotation direction). This allowed an analysis of whether cases of right or left visual motion impacted results. The data showed there was no left/right direction difference and these directional conditions were eventually collapsed to examine whether cases of congruent or incongruent head rotations had differential effects on visual motion perception, with the stationary condition serving as a control.

### Results

#### Incongruent head rotations increase visual motion detection thresholds

**Figure 2.**
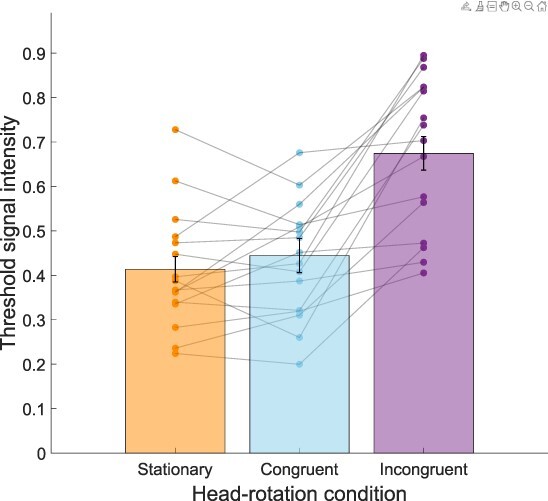
Average thresholds for head rotation conditions (collapsed across left/right stimulus motion directions). Each dot is a participant (participants with missing data not shown) and columns show group means with error bars showing standard error of the mean. An ANOVA and LMM both showed a significant difference in motion thresholds based on head-rotation condition. Post-hoc comparisons revealed significantly higher thresholds in the incongruent than congruent and stationary conditions. *** denotes *P <* 0.001, Bonferroni corrected

This experiment investigated motion perception thresholds in the presence of congruent, incongruent, or no simultaneous head rotation. Using a 2 × 3 (left/right visual motion, congruent/incongruent/stationary head rotation) repeated-measures analysis of variance (ANOVA), visual motion direction (left or right) was found to have no significant influence on thresholds for visual motion perception (*F*(1,7) = 5.29, *P =* 0.06). There was a significant main effect of rotation congruence on thresholds (*F*(2,14) = 18.04, *P <* 0.001, η^2^_p_ = 0.72), with no interaction (*F*(2,14) = 3.64, *P =* 0.06; Fig. 2). Post-hoc pairwise comparisons with Bonferroni correction showed that incongruent visual-active vestibular motion signals resulted in significantly higher thresholds than both congruent or stationary conditions (*t*(14) = 5.11, *P <* 0.001, *d *= –1.37 and *t*(14) = 5.29, *P <* 0.001, *d* = 1.42, respectively). An LME was also run to quantify the strength of this main effect when modelling the contribution of individual participant data as a random effect. Likelihood ratio tests confirmed that including Congruency as a fixed effect significantly improved model prediction compared to the basic model with only random effects per participant (χ^2^(2) = 28.09, *P* < 0.001). There were specific fixed effects for congruent compared to stationary (β = –0.07, 95% CI = [−0.13, −0.02], *t*(95) = −2.59, *P =* 0.011), incongruent compared to stationary (β = 0.16, [0.10, 0.22], *t*(95) = 5.80, *P <* 0.001), and congruent compared to incongruent (β = 0.23, [0.13, 0.33], *t*(95) 4.73, *P* <0.001). Including Direction did not significantly improve any models, nor did the inclusion of the interaction term (Congruency * Direction).

#### Speed of head rotation

**Figure 3.**
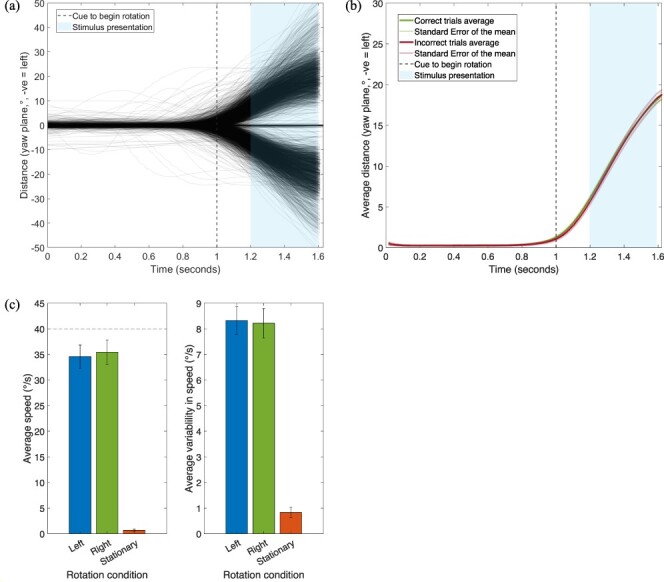
(a) Head rotation for each rotation trial of every participant. The cue to begin the turn came from the head guide (travelling at 40°/s) reached a centre marker. Trials with erroneous rotations were removed before analysis. Red represents trials that had incorrect responses, green trials with correct direction judgements. (b) Average absolute value of trajectories across participants for trials that were correct or incorrect. Shaded lines represent SEM. (c) Average speed and variability of head rotations across each participant, error bars represent SEM. Average variability was calculated by taking the mean standard deviation in speed across participants. A dashed line at 40°/s indicates speed of head guide

We analysed participants’ ability to follow the head guide and any potential effects of head-speed on visual motion detection thresholds. One participant was found to have not turned their head in rotation conditions and made significant movements while stationary, so was removed from analysis. Participants were on average slightly slower than the guide but consistent between both rotation conditions. Figure 3a shows the specific time course of head rotation, including when stimuli were presented. Figure 3b shows the average absolute rotation across all participants, separated by trials when participants correctly or incorrectly judged motion direction. Figure 3c shows the average speeds and variability for each rotation condition. These averages could be slightly lower than the target speed as they were calculated over the entirety of the rotation, including the initial acceleration and ending deceleration periods. When fitting a linear model to each participant’s average speed and threshold value when turning left and right, there was no significant correlation between rotation speed and threshold (right, *R*_Adj_^2^ = –0.002, *F*(1,16) = 0.96, *P =* 0.34; left, *R*_Adj_^2^ = 0.11, *F*(1,17) = 3.25, *P =* 0.09). This suggests that differences in head speed rotation did not drive any difference in thresholds we report. It is clear participants could follow the guide close to the target speed and with minimal variability with angular velocity during the presentation of the stimulus was close to constant. This gives confidence that the current method of following a head guide in a VR scene is a valid method of studying the influence of active head rotations on visual motion perception.

#### Head rotation direction biases perceived motion direction

**Figure 4.**
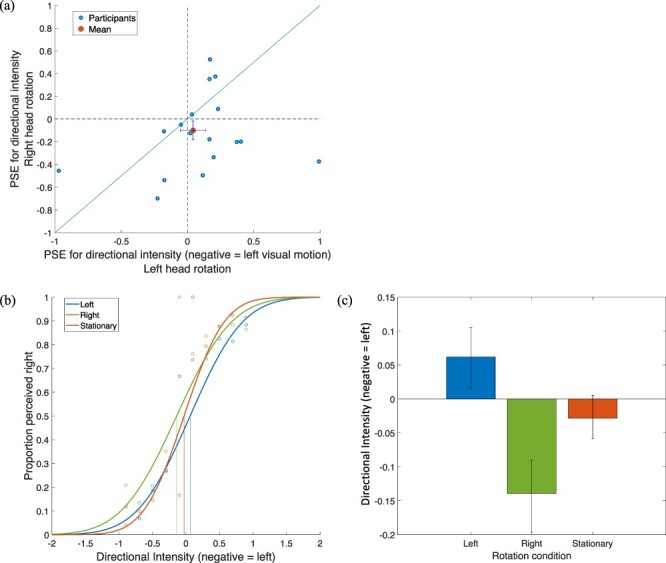
Directional intensity refers to the threshold signal intensity of the stimulus, usually between 0 and 1, however adapted here to additionally indicate the direction of motion present in trials (left or right). (a) PSEs for directional intensity with left visual motion coded as negative. Threshold values for leftward-rotation trials for each subject were plotted against the corresponding threshold values for rightward-rotation trials and the dashed line shows the equality line. The group mean is off the diagonal in the lower half and suggests a bias to make visual motion responses consistent with the direction of head rotation, as the likelihood of indicating rightward perceived motion is not equal between cases of rotating right and left. Participants with missing data not shown. (b) PMFs generated for a super subject showing a shift in average threshold value for each rotation condition. (c) Average threshold value for each rotation condition after super subject data were bootstrapped 1000 times. Error bars show 95% confidence intervals

In addition to the condition-average effects (see Fig. 3), we additionally calculated the point-of-subjective equality (PSEs) after fitting psychometric functions (PMFs) to the motion signal intensities of each condition (see Methods). For this analysis, signal intensities were re-coded to be negative if the signal direction was leftward and positive if rightward. PMFs were then fitted for each participant to show responses as percentages of rightward responses, with leftward and rightward threshold values plotted against each other, as shown in Fig. 4a. As the average PSE seemed to cluster off the diagonal, potentially signifying a bias in participant responses, we performed a super subject analysis to investigate the presence of this bias in our sample. Figure 4b shows the PMFs generated for the super subject for each rotation condition. The statistical reliability of these differences in super subject thresholds was investigated via a bootstrapping procedure using full resampling of the data with replacement. Data were bootstrapped 1000 times and mean bootstrapped thresholds are shown in Fig 4c. Error bars represent 95% CIs, suggesting there is a significant bias for participants to answer in line with the direction of head rotation.

Together, these results indicate a cost imposed upon motion detection thresholds by incongruent visual motion/head rotation such that thresholds are higher for detection in incongruent compared to congruent or stationary conditions. In addition, a bias to respond to left/right motion congruently with head-rotation was observed, and became the focus of Experiment 2.

## Experiment 2

Experiment 1 found that incongruent visual-active vestibular motion impaired motion detection thresholds. Interestingly, participants also exhibited a significantly increased likelihood to report left or right perceived motion when performing the same direction of head-turn. If participants were biased to respond in line with the direction of head turn, this could explain the lowered threshold for congruent visual motion, as it would have driven a higher rate of correct responses for congruent trials. We cannot be sure then whether the difference in congruent and incongruent thresholds in Experiment 1 reflects differential processing of signals or this response bias (or some combination of both). In Experiment 2 we removed the mapping of left/right response options in a revised version of our task to investigate this bias to report congruent motion in the absence of overlapping left/right response criteria.

### Methods

#### Participants

Twelve participants were recruited from the University of Sydney undergraduate Psychology cohort, and one was removed for failing to achieve 75% accuracy. The remaining 11 had corrected-to-normal vision and no neck or vestibular conditions were naïve to the purposes of the experiment and gave informed consent prior to participation. The study protocol was approved by the University of Sydney Human Research Ethics Committee (HREC 2021/048).

#### Stimuli

**Figure 5.**
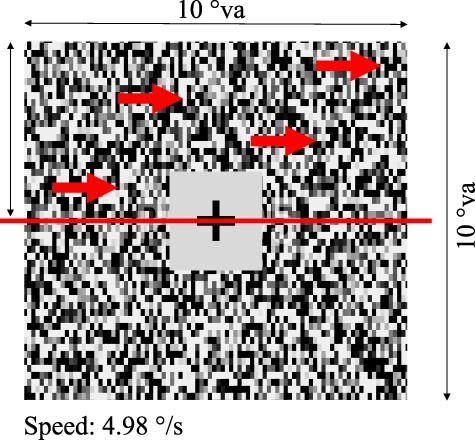
Stimulus for Experiment 2. The motion signal was only presented in the upper or lower half (with the other half stationary). The task became a two-alternative forced-choice: which half (upper or lower) contained the motion? This removed the possibility of response bias and isolated sensitivity to the motion task. The dissecting line, and arrows, are for illustration purposes only and were not present in the actual experiment

The motion signal was generated following the same method as in Experiment 1. The aperture occupied 10.00 × 10.00 °va. To aid fixation the stimulus contained a central inner grey square and fixation cross, occupying 2.29 and 1.17 °va, respectively. In this experiment, however, the stimulus was split into an upper and lower half, with the motion occurring in one half only, again moving either leftward or rightward (see Fig. 5). Participants responded using the up or down arrow keys to indicate which half of the stimulus (upper or lower) contained the motion, regardless of its direction. The task thus emphasized motion detection.

#### Procedure

Procedure for motion cueing was the same as experiment 1 (following a head guide). Judged from head rotation data in Experiment 1, the presentation of the stimulus in the current experiment was delayed slightly to better ensure the stimulus was viewed during constant angular velocity. The stimulus was therefore presented 1.2 s after the arrival of the motion guide with 400 ms duration. Participants indicated which half of the stimulus contained the visual motion using the up and down arrow keys. Participants completed a practice version of the experiment to get used to following the head guide and responding to the stimulus. The practice experiment consisted of 30 trials (10 stationary, 10 with congruent motion and 10 with incongruent motion, with equal split of motion travelling left or right within each condition). In the experiment proper, each participant completed two back-to-back sessions of 180 trials each (2 stimulus directions × 3 head rotations x 30 trials per condition) giving a total of 360 trials per participant. Presentation of the stimulus in the upper or lower hemifield was equally distributed in each condition. All trial types were presented in a random order within each session. A Quest adaptive staircase controlled the stimulus intensity for each trial to maintain 75% correct performance.

### Results

#### All head rotations increase visual motion detection thresholds

**Figure 6.**
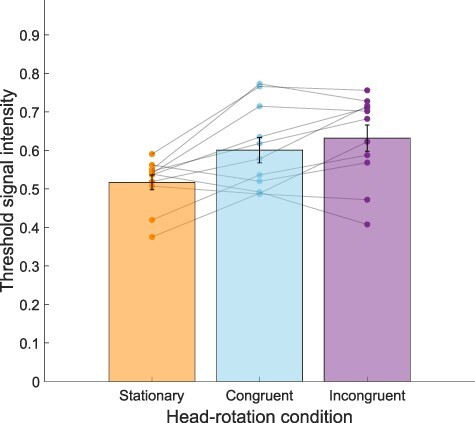
Average thresholds for visual-active vestibular motion conditions (collapsing across left/right motion direction). A repeated-measures ANOVA with post-hoc comparisons indicated motion thresholds when stationary were significantly lower than during both congruent and incongruent head rotations. Dots indicate individual participants, columns show the group mean with error bars showing standard error of the mean. * denotes *P <* 0.05, *** *P <* 0.001

A 2 × 3 repeated-measures ANOVA (stimulus motion vs head rotation) showed that visual motion direction (left/right) did not significantly affect motion-detection thresholds (*F*(1,9) = 0.26, *P =* 0.62). In contrast, a significant main effect of head congruency was observed (*F*(2,18) = 10.68, *P <* 0.001, η^2^_p_ = 0.54). The interaction between congruence and direction was also non-significant (*F*(2,18) = 2.24, *P =* 0.14). Post-hoc pairwise comparisons (Bonferroni corrected) showed both congruent and incongruent conditions resulted in significantly higher thresholds than the stationary condition (*t*(9) = 3.24, *P =* 0.01, *d *= 1.03 and *t*(9) = 4.47, *P <* 0.001, *d *= 1.41, respectively). Unlike in Experiment 1, congruent and incongruent conditions were found not to differ significantly from each other (*P =* 0.70, see Fig. 6).

We performed an additional linear mixed effects analysis to confirm our main results while accounting for the influence of individual participants as random effects. Including Congruency significantly improved the model fit compared to the basic model with random effects for participants (χ^2^(2) = 16.28, *P* <0.001). There were specific fixed effects for incongruent compared to stationary (β = 0.05, [0.02, 0.08], *t*(60) = 3.11, *P =* 0.002), but not congruent compared to stationary (*P =* 0.27), or congruent compared to incongruent (*P =* 0.25). Once again, including a term for Direction did not improve upon this model (χ^2^(1) = 0.58, *P* = 0.44), nor did a model with their interaction (χ^2^(3) = 3.49, *P* = 0.32).

#### Speed of head rotation

**Figure 7.**
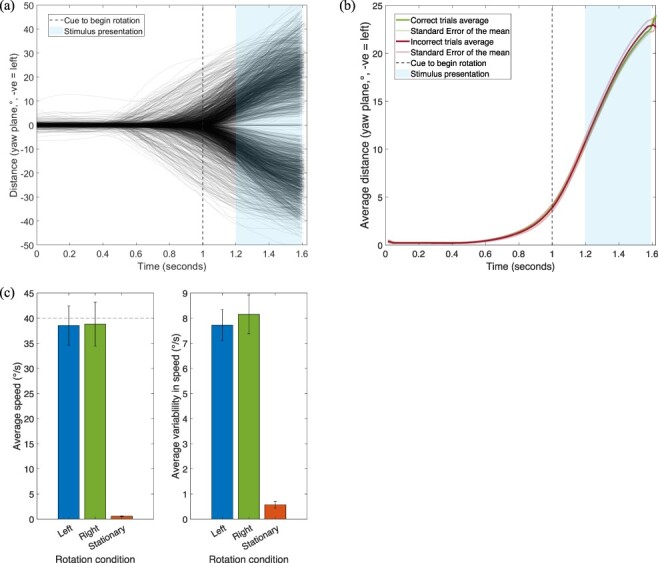
(a) Head rotation for each rotation trial of every participant. The cue to begin the turn came from the head guide (travelling at 40°/s) reached a centre marker. Trials with erroneous rotations were removed before analysis. (b) Average absolute value of trajectories across participants for trials that were correct or incorrect. Shaded lines represent SEM. (c) Average speed and variability of head rotations, error bars represent SEM. Average variability was calculated by taking the mean standard deviation in speed across participants. A dashed line at 40°/s indicates speed of head guide

We analysed participants’ ability to follow the head guide and any potential effects of rotation speed on visual motion detection thresholds. As in Experiment 1, Fig. 7 clearly shows participants’ ability to follow the head guide accurately and precisely. A linear model showed when turning right, there was no significant correlation between rotation speed and threshold (*R*_Adj_^2^ = 0.015, *F*(1,9) = 1.15, *P =* 0.31). The same was found for leftward head rotations (*R*_Adj_^2^ = 0.14, *F*(1,9) = 2.67, *P =* 0.14).

## Discussion

In this study, we tested visual motion detection thresholds in the presence of congruent and incongruent head rotation. In two experiments, participants were tasked with performing brief rotational head movements in a VR environment and to indicate whether the retinal motion of a stimulus presented in a fixation-centred display was moving leftward or rightward (Experiment 1) or whether motion (in either direction) was present in the upper or lower hemifield (Experiment 2). We discovered elevated motion thresholds for active head rotations, in particular during incongruent visual-active vestibular stimulation.

Analysis of subjects’ ability to follow the head guide across both experiments showed participants, while on average a little slower (but not significantly) in turning their head than the speed of the guide, can accurately and precisely follow the guide to perform active head rotations. This validates the current method as a viable paradigm for studying the influence of head rotation on visual motion perception. In addition, the ability to track the entire course of each rotation allows analysis of the true speed of each participant, if desired.

The increased motion threshold during head rotation may result from an increased likelihood of attributing motion signals to self-motion during incongruent head rotations, owing to a world-stationarity prior (Weiss et al. 2002, Welchman et al. 2008, Freeman et al. 2010). Specifically, as most incongruent visual motion signals are frequently attributed to self-motion in everyday activities, this strong prior could result in significantly higher thresholds for perceiving incongruent visual motion, as found in Experiment 1. This supports the hypothesis that visual motion viewed while turning the head in an opposite direction must be stronger than when viewed while turning in a congruent direction (or remaining stationary), in order to overcome the bias to perceive such visual motion as ‘normal’ scene-shift resulting from self-motion (Weiss et al. 2002, Welchman et al. 2008, Freeman et al. 2010). This could be driven by cells tuned selectively for incongruent visual-vestibular rotation in MSTd (Takahashi et al. 2007). However, participants were also found to have a bias to report the same direction of motion as their head rotation, potentially resulting in a higher rate of incorrect responses for cases of incongruent motion, and a higher correct rate for congruent motion. To test this, Experiment 2 used a two-alternative forced-choice task to remove this potential response bias to better determine whether the results were driven by low-level differences in visual motion sensitivity or by a response-level directional bias. Interestingly, the motion threshold results showed that congruent head rotations did not produce better performance than incongruent rotations. This finding can be contrasted with results of analogous eye tracking studies which show that smooth pursuit eye movements produce a benefit for the tracked stimulus (e.g. Greenlee et al. 2002, Spering et al. 2011). We suggest that functional benefit may not apply to tracking achieved via active head rotation once response confounds are controlled to isolate threshold sensitivity.

### Self-motion bias in visual motion perception

The demonstration of a self-motion bias is intriguing and could represent a low-level bias that upweights vestibular cues when visual stimuli are close to threshold in an attempt to gain a more reliable percept of veridical motion after multisensory integration (Ernst and Banks 2002, Ernst and Bülthoff 2004, Morgan et al. 2008). On the other hand, the fact that participants were biased to report the direction of head rotation could also be explained by a higher-level response bias to answer in line with head rotation when stimulus uncertainty is high. This is supported by other findings of correlated heading and object motion biases (Dyde and Harris 2008, Wu et al. 2021, Xing and Saunders 2022). For example, Xing and Saunders (2022) found that perceived heading based on both visual and vestibular cues positively correlated with judgements of object motion, akin to the correlated rotation-direction decisions in this study. Dyde and Harris (2008) found that for an object to appear stationary, it had to move with the motion of an observer, to counteract the effect of observers perceiving the object to be moving away from them when truly earth stationary. This could be an example of an incorrect accounting of motion signals, which could also be driving the higher rotational thresholds in the current experiment.

In other related work, Wu et al. (2021) used context trials of random-dot kinematograms (RDK) travelling in a certain direction within a trial block to build an expectation of direction. When shown a low-coherence RDK and asked to indicate perceived direction, participants showed a perceptual repulsion bias such that direction judgements were made in a direction opposite the expected direction. If an opposite visual signal is expected when a rotation of the head is made, then a similar repulsion bias in the current experiment would result in greater numbers of responses in the direction of the head turns. These prior findings could explain the bias in the current experiment and the subsequent differences in threshold for congruent and incongruent conditions. Further, Wu et al. (2021) showed by manipulating the coherence of a motion signal in an RDK that the repulsive effect was not due to low-level adaptation but rather to a perceptual bias. It was also present when eyes were fixated so also could not be attributed to eye movements themselves but rather a system that prioritizes processing of novel or ‘unexpected’ stimuli. The perceptual bias and priority processing supports the favoured explanation of the current results. Namely, if oppositely tuned visual-vestibular rotation cells in the MSTd suppress the expected incongruent motion of a shifting stationary scene due to self-rotation, an ‘unexpected’ congruent motion signal, which is more likely therefore to signal object motion, could be more easily perceived. This is supported by Alais et al.’s (2021) findings that during binocular rivalry between competing leftward and rightward visual motions, the direction congruent with the direction of active or passive self-rotation is perceived. Priority processing of congruent visual motion is also consistent with findings of a vestibular boost of visual motion processing (Hogendoorn et al. 2016, 2017).

It is therefore unclear whether a low-level physiologically driven perceptual effect or higher-level directional bias accounts for the results in Experiment 1. In an attempt to at least partially answer what is the driving force behind these results, Experiment 2 decoupled the perception of motion from direction judgement, so the possible low-level effects of visual-active vestibular motion perception could be explored. In Experiment 2, instead of responding with a left–right arrow press to indicate perceived direction, participants indicated whether they detected motion at all, regardless of leftward or rightward direction, in the upper or lower half of the stimulus. Interestingly, this method showed no significant difference between motion thresholds for incongruent and congruent head turns, and both were significantly higher than thresholds for viewing motion while stationary. This pattern of results lends favour to bias driving the differences between congruent and incongruent thresholds observed in Experiment 1. Moreover, the finding that motion thresholds were elevated in a way that was not selective for visual motion/head rotation congruence supports the idea that it might arise from increased attentive, cognitive, or task demands associated with active movements and thus elevated thresholds (Lavie et al. 2004, Swallow and Jiang 2013, Heinrich et al. 2020). Specifically, requiring participants to follow a head guide while also judging stimulus motion direction could be considered a dual-task paradigm, which is known to have performance costs (Bourke et al. 1996, Lavie et al. 2004), especially for dual-tasks of common modalities as in the current case (Bourke et al. 1996).

### Smooth-pursuit comparisons

It was hypothesized that congruent head rotations in the current experiment could mirror results of MacNeilage et al. (2012), where object discrimination thresholds were significantly lower when accompanied by congruent vestibular cues, compared to just visual cues from optic flow. However, no such benefit was found in Experiment 2 as congruent and incongruent rotations showed no significant difference for visual motion detection thresholds, and both were significantly increased from stationary. An explanation might be found in smooth-pursuit eye-movement literature. While such movements have been found to aid perception of coherent visual motion when compared to fixation (Greenlee et al. 2002) in addition to direction and speed judgements, lowering perceived motion smear, and improving motion coherence (see Spering and Montagnini 2011 for a review), smooth pursuit has also been found to worsen motion perception (Spering and Montagnini 2011), with two well-known examples being the Filehne (Mack and Herman 1973) and Aubert-Fleischl phenomena (Dichgans et al. 1975), where direction and speed judgements, respectively, are impaired, again in comparison to fixation. More recently, Arathorn et al. (2013) found whenever retinal image motion was opposite to eye motion; the perception of the relative motion was reduced. Finally, Krukowski et al. (2003) found no difference in direction perception between fixation and smooth pursuit conditions.

It seems then that current results from tracking achieved via active head rotation may align more closely with findings suggesting perceptual costs of smooth-pursuit eye movements, rather than those reporting perceptual benefits. Whether this is due to the active nature of the movement, with potentially noisy motor and proprioceptive signals to be processed in addition to vestibular signals, or a worse ability to account for vestibular signals when processing visual stimuli, remains unknown without a passive case comparison.

### Active vs passive perception

The difference in ability to accurately perceive visual motion while rotating the head compared to the head-stationary condition could also signal a general effect of imperfect estimates of the self-motion signal (Dyde and Harris 2008, MacNeilage et al. 2012, Dupin and Wexler 2013, Garzorz et al. 2018, Britton and Arshad 2019, Xing and Saunders 2022, Zeng et al. 2023). In essence, robust visual motion perception during self-motion relies on accurate accounting of such self-motion so that object motion can be disambiguated and veridically perceived. It is well-theorized that this calculation relies on an efference copy of a motor command being used to predict the sensory outcome, with the prediction then used to subtract motion generated by self-motion from the incoming signal (reafference), leaving only motion generated from external sources (exafference) to be perceived (Roy and Cullen 2001, Cullen 2012). In this study, this would mean trials requiring a head rotation would generate an expected visual signal in an opposite direction to the head turn, which in an ideal system would be accounted for when assessing the presence of any exafferent motion. However, it is widely thought that this accounting is not perfect, and resulting errors in motion perception are common (Dyde and Harris 2008, MacNeilage et al. 2012, Dupin and Wexler 2013, Garzorz et al. 2018, Britton and Arshad 2019, Xing and Saunders 2022, Zeng et al. 2023). These errors are thought to arise from an incorrect subtraction of eye-movement from retinal motion, resulting in an over or under estimation of target motion (Rushton and Warren 2005, Dupin and Wexler 2013). In particular, Swanston and Wade (1988) found pursuit movements of the head were less effectively compensated for than movement of the eyes. Our eyes are specialized to make fast, precise movements, whereas our heads are much heavier and would take a greater effort to turn. This increased resistance could lead to greater variability in head rotations which in turn could increase the variability in the theorized subtraction process for veridical motion perception. Further, high variability can lead to an increased reliance on priors, which as discussed above, would in the current case translate to an increased expectation for the world to be stationary (Weiss et al. 2002, Welchman et al. 2008, Freeman et al. 2010). These fundamental differences could explain why congruent rotations of the head did not provide any benefit to perceiving visual motion, in contrast to what was predicted based on findings in smooth pursuit eye movements.

Sensory reafference is only possible in cases of active self-motion, as passively stimulating the vestibular system would not generate any prior motor commands. Active perception also involves proprioceptive signals for self-motion, in addition to vestibular ones and without comparison to a passive case, disambiguating the effect of each component is impossible. Since vestibular nuclei show a reduced responsiveness to vestibular afferent signals when motion was generated by an active head turn (Roy and Cullen 2001), investigating the effect of active vs passive interactions on visual motion thresholds should be a key focus for future studies. For example, a motion platform could be used to passively rotate an observer at a rate matching the active head rotations used in these experiments and thus isolate the vestibular contribution to the effects reported here. It is important to differentiate between cases of active and passive perception as a growing body of literature is finding that active movement can have significant effects on our sensory functions (Alais et al. 2021, Davidson et al. 2023).

### Foreground vs background motion

Another limitation of this study was the fact that the depth of the visual stimulus was somewhat ambiguous. Swanston and Wade (1988) suggest that different to eye movements, head movements need a 3D compensation that accounts for perceived egocentric distance of an object of interest, so judgements of object distance could affect compensation of self-motion when judging object motion. If different participants were perceiving the current stimulus at different depths, perception of visual motion could have been impaired and thus masked any underlying effect of congruence. Dupin and Wexler (2013) highlight that motion of a scene’s background does affect perceived motion of a foreground object. Dyde and Harris (2008) found that errors in depth, while not fully explaining impairment in motion perception, do influence results. In addition to this literature, it follows common sense that our motion processing system might treat differentially motion of the foreground or background, given the background as a whole is more likely to be stationary, causing an opposite visual motion signal to self-rotation. On the other hand, objects in the immediate foreground of a scene may be more likely to be attached to the body in some way (e.g. a tea cup being carried) and therefore generate motion signals congruent with the body. Either way, object motion would not take up the entire scene like a scene-shift signal would, and therefore could be highlighted as a potential object of interest. Future studies should investigate the potential differential processing of congruent and incongruent self-motion signals on perceptual thresholds for foreground and background motion.

## Conclusion

The direction of active rotational head movements do not appear to influence visual motion thresholds other than to increase thresholds when compared to viewing motion while stationary. These findings contrast with prior research on the advantage of tracking and smooth-pursuit eye movement. It is recommended that future research incorporate the active and passive generation of vestibular signals to develop a greater understanding of the role of rotational self-motion on visual motion perception.

## Data Availability

The data underlying this article will be shared on reasonable request to the corresponding author.
